# From odor to order: unveiling the crucial role of hydrogen sulfide in plant life

**DOI:** 10.1093/hr/uhaf273

**Published:** 2025-10-17

**Authors:** Zhuping Jin

**Affiliations:** Shanxi Key Laboratory for Research and Development of Regional Plants, School of Life Science, Shanxi University, Taiyuan, Shanxi Province 030031, China

## Abstract

Hydrogen sulfide (H_2_S) has garnered significant attention as a novel gaseous signaling molecule. While its physiological roles in animals are well documented, research over the past two decades has increasingly uncovered its vital functions in plants, establishing it as a crucial component in plant signaling processes. In plants, endogenous H_2_S is produced across various subcellular compartments and plays indispensable roles in stress responses, growth, and development. Research has progressed from model plants to horticultural crops, underscoring the prospective agricultural benefits of H_2_S. Nevertheless, several challenges persist, including unclear signaling targets and limited real-world applications. This comprehensive review explores the discovery, biosynthesis, physiological roles, mechanisms, and molecular targets of H_2_S in plants, offering valuable insights in future research.

## Introduction

Gases such as nitric oxide (NO), carbon monoxide (CO), and hydrogen sulfide (H_2_S), once regarded as toxic, are now recognized as gasotransmitters with significant physiological functions in both animals and plants [1, 2]. This shift highlights evolutionary adaptations and the sustained utilization of primitive atmospheric molecules by living organisms [2, 3]. Notably, H_2_S, identified as a gasotransmitter nearly two decades ago, regulates diverse physiological processes in plants, including growth enhancement, stress mitigation, photosynthesis, flowering, and crop quality improvement [4–6]. Numerous reaction-based assays have been developed to detect H_2_S concentrations *in vivo* and *in vitro*. These findings have been applied to horticultural practices, especially in the production of vegetables, fruits, and ornamentals [7]. However, key obstacles remain in the practical manipulation H_2_S signaling in field conditions, such as the development of sustained-release formulations, maintaining efficacy in open environments, determining optimal doses for different crops and growth stages, and refining application techniques for specific objectives.

## Physiological functions of H_2_S signaling in plants

**Figure 1:**
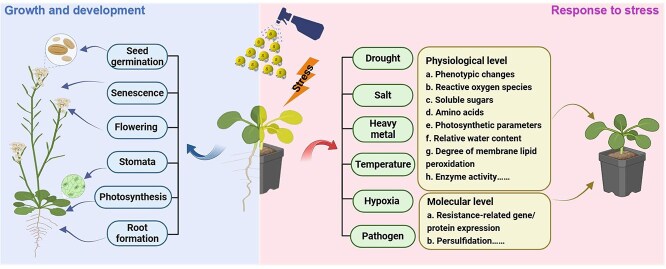
A model of the physiological effects of H_2_S in plants.

Scientists are gradually uncovering the physiological functions of H_2_S through experiments involving the use of various donors, inhibitors, and scavengers targeting specific H_2_S-mediated processes (Fig. 1).

### H_2_S promotes plant growth and development

H_2_S is essential throughout plant development, from seed germination to senescence. It promotes the production of the nuclear protein CONSTITUTIVE PHOTOMORPHOGENIC 1 (COP1), which leads to the degradation of the ELONGATED HYPOCOTYL 5 (HY5) transcription factor and the downregulation of the basic leucine zipper transcription factor ABA (abscisic acid)-INSENSITIVE 5 (ABI5), thereby enhancing seed germination rates under heat-stress conditions [8, 9]. Furthermore, H_2_S signaling initiates cyanide-resistant respiration, further accelerating germination [10]. H_2_S also influences root morphogenesis in a dose-dependent manner [11, 12]. H_2_S increases the accumulation of reactive oxygen species (ROS) and hydrogen peroxide (H_2_O_2_), and upregulates the expression of respiratory burst oxidase homolog 1 (RBOH1), a plant-specific NADPH oxidase that plays a central role in ROS production, facilitating lateral root formation in tomatoes [13]. Additionally, H_2_S modulates ACTIN dynamics and supports root hair growth [14, 15]. Exogenous H_2_S application, especially at a concentration of 100 μM, significantly improves leaf photosynthetic efficiency by regulating the transcription and enzymatic activity of ribulose 1,5-bisphosphate carboxylase/oxygenase (RuBisCO) [16, 17]. Persulfidation is now recognized as a key redox regulatory mechanism in H_2_S signaling [18]. As the plant progresses to the flowering stage, H_2_S reduces promoter binding activity via persulfidation of BraFLC and modulates gene splicing related to flowering through the persulfidation of AtU2AF65a, ultimately promoting early flowering [19, 20]. H_2_S also plays a pivotal role in plant senescence, functioning as a regulatory molecule in various vegetables and fruits [21, 22]. In tomato seedlings, *SlLCD1*, which encodes a nuclear-localized cysteine desulfhydrase enzyme essential for endogenous H_2_S production, triggers premature leaf senescence in knockout mutants, whereas its overexpression in transgenic lines delays senescence compared to the wild type [23]. H_2_S boosts biomass production and delays senescence in rhizobium-colonized soybean by increasing nodule number, chlorophyll content, nitrogen uptake efficiency, and tissue nitrogen concentration [24]. Studies have shown that declining persulfidation levels in aging nodules disrupt redox balance, promoting senescence [25]. Beyond its role in growth and senescence, H_2_S also prevents tomato flower abscission by restoring the basipetal auxin gradient [26, 27].

### H_2_S improves stress response in plants

H_2_S enhances plant responses to a range of abiotic and biotic stresses. Acting as a signaling molecule, it improves drought tolerance in *Arabidopsis* by interacting with ABA to regulate stomatal behavior [28, 29]. This enables plants to respond more effectively, with protein persulfidation playing a key role in managing drought-induced stress [6, 30]. In addition, H_2_S treatments enhance gene expression and the phosphorylation of plasma membrane H^+^-ATPase (PMA) and Na^+^/H^+^ antiporter proteins, while maintaining lower Na^+^/K^+^ ratios, thereby increasing salt tolerance [31]. Further research found that H_2_S induces the persulfidation of PMA1, activating the protein and promoting its interaction with GENERAL REGULATORY FACTOR 4, which further enhances salt tolerance in *Arabidopsis* [32]. H_2_S also improves heat tolerance in maize seedlings [33]. Under high-temperature conditions, H_2_S signaling elevates nuclear COP1 levels, resulting in HY5 protein degradation and reduced *ABI5* expression, thereby enhancing heat tolerance during seed germination [9]. H_2_S significantly improves tolerance to heavy metal stress, particularly cadmium (Cd), a phenomenon that has been widely studied [34–36]. Modeling of rice–Cd interactions has shown that under Cd stress, H_2_S reduces Cd uptake and accumulation, elevates mineral nutrient content, enhances photosynthetic performance, and rapidly activates the antioxidant defense system [37]. Additionally, H_2_S alleviates nickel-induced growth inhibition in both roots and aerial parts of zucchini plants [38]. This protective effect may involve crosstalk between calcium ions (Ca^2+^) and H_2_S. H_2_S mitigates zinc toxicity by lowering H_2_O_2_ levels, reducing electrolyte leakage, and malondialdehyde concentrations (a byproduct of ROS, ROS-induced membrane damage), while enhancing antioxidant enzyme activity and nutrient uptake [39]. In maize exposed to lead-contaminated environments, H_2_S increases nitrate reductase activity and glutathione content, regulates amino acid profiles, promotes growth, and reduces lead accumulation [40]. H_2_S also strengthens maize tolerance to hypoxia induced by NO [41]. Furthermore, H_2_S enhances resistance to biotic stresses by inhibiting fungal growth, spore germination, and mycelial development on fruits, thereby improving overall plant disease resistance [42, 43].

Through the coordinated action of two guard cells, stomata regulate gas and water vapor exchange, enabling plants to adapt to changing environmental conditions. H_2_S enhances drought avoidance by inducing stomatal closure under water-stress conditions [28] and modifies stomata-associated proteins through persulfidation [44, 45]. H_2_S-induced stomatal closure has been linked to energy metabolism, with the mitochondrial H_2_S donor 10-(4-carbamothioylphenoxy)-10-oxodecyl triphenylphosphonium bromide (AP39) regulating mitochondrial function in guard cells to promote closure [46]. This process partly relies on succinate dehydrogenase (SDH), a key component of the mitochondrial electron transport chain [44]. H_2_S also triggers stomatal closure by modulating ion channel activity [47–50] and is recognized as a critical regulator of stomatal closure under various environmental stresses, influenced by phytohormones and signaling molecules such as ABA [29, 51, 52], H_2_O_2_ [53], and ethylene [54]. Additionally, H_2_S influences stomatal development [55–57], further highlighting its role in enabling plants to adapt to environmental changes. Further research is needed to clarify the specific function of mitochondrial H_2_S synthase in stomatal immunity, the primary gateway of the plant pathogen defense system [58, 59].

## The relationship between H_2_S and other signals

**Figure 2:**
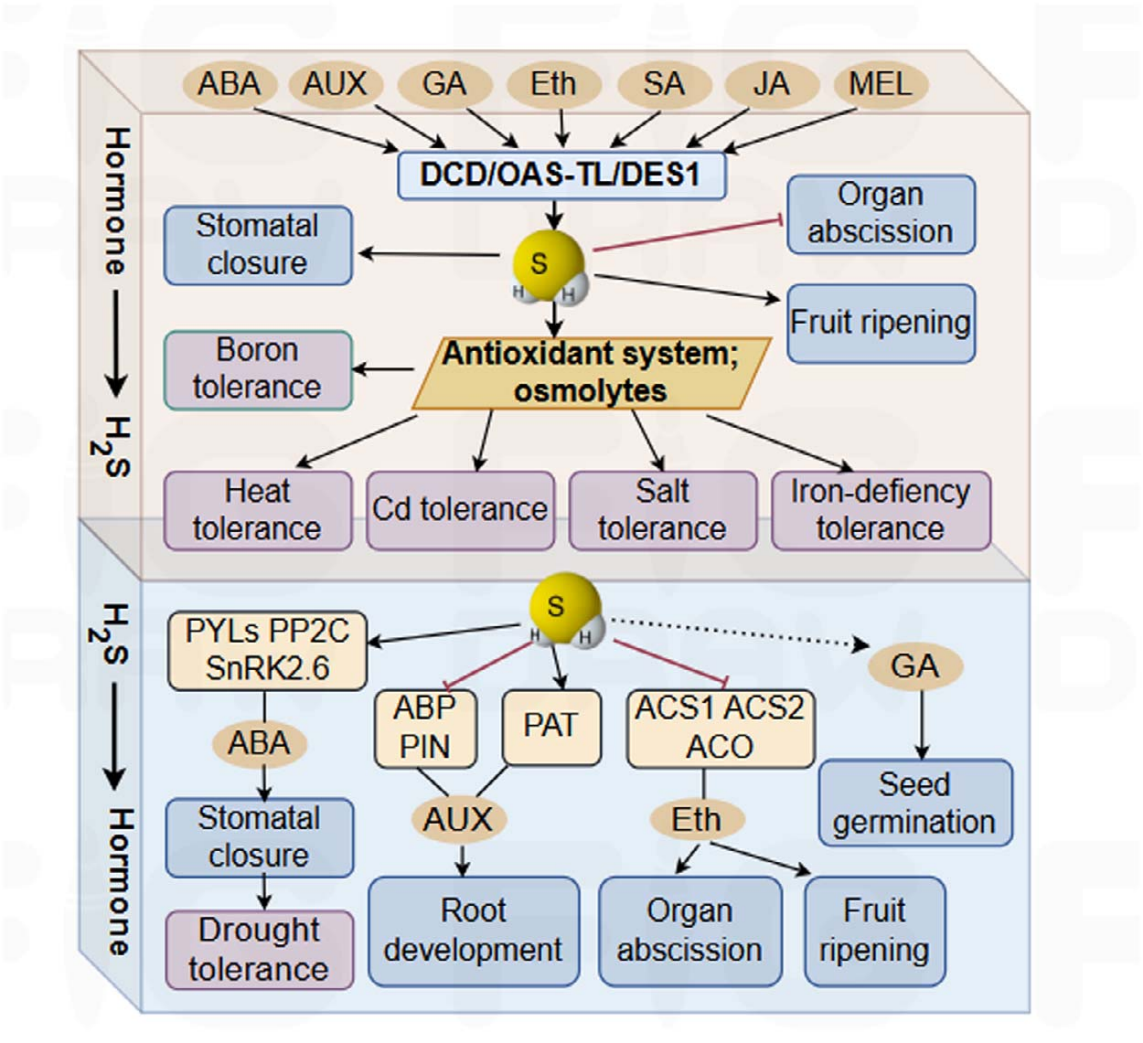
Interaction between H_2_S and plant hormones. Arrow end: activation; blunt end: inactivation. ABA, abscisic acid; ABP, actin-binding protein; ACO, ACC oxidase; ACS, ACC synthase; AUX, auxin; DCD, D-cysteine desulfhydrase; DES1, desulfhydrase; Eth, ethylene; GA, gibberellin; H_2_S, hydrogen sulfide; JA, jasmonic acid; MEL, melatonin; OAS-TL, *O*-acetylserine(thiol)lyase; PAT, peroxisomal ABC (ATP-binding cassette) transporter; PIN, polar auxin transport carrier protein; PP2C, protein phosphatase type 2C; PYLs, pyrabactin resistance/pyr-like; SA, salicylic acid; SnRK2.6, SNF1-related serine/threonine-protein kinase.

**Figure 3:**
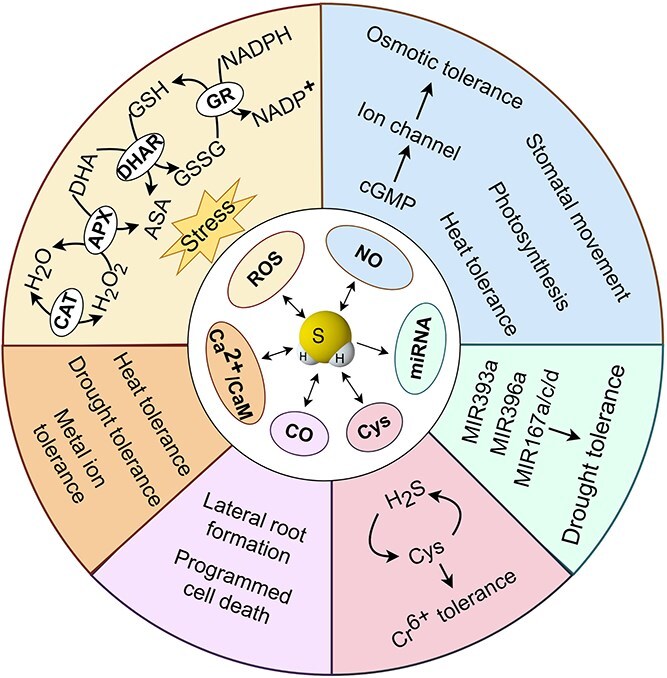
Interaction between H_2_S and other molecules. Arrow end: activation; blunt end: inactivation. APX, ascorbate peroxidase; ASA, acetylsalicylic acid; CaM, calmodulin; CAT, catalase; cGMP, cyclic guanosine monophosphate; CO, carbon monoxide; Cys, cysteine; DHA, docosahexaenoic acid; DHAR, dehydroascorbate reductase; GSH, glutathione; GR, glutathione reductase; GSSG, glutathione disulfide; H_2_O_2_, hydrogen peroxide; H_2_S, hydrogen sulfide; NADPH, nicotinamide adenine dinucleotide phosphate; NO, nitric oxide; ROS, reactive oxygen species.

H_2_S also maintains a complex interplay with other signaling molecules involved in regulating plant growth, development, and stress responses (Figs 2 and 3).

### H_2_S and phytohormones

The physiological processes of higher plants are governed by an intricate signaling network involving plant hormones. H_2_S modulates hormonal balance, thereby influencing both growth and stress tolerance [60].

ABA, often referred to as the stress hormone, plays a central role in regulating plant tolerance to abiotic stresses. The interaction between H_2_S and ABA, particularly in relation to stomatal regulation, has been extensively studied. H_2_S induces stomatal closure and participates in ABA-dependent signaling, likely by modulating ABC transporter proteins in guard cells [61]. Exogenous ABA significantly elevates leaf H_2_S levels and enhances the activities of both L-cysteine desulfhydrase (LCD) and D-cysteine desulfhydrase (DCD) [62], while the application of the H_2_S scavenger hypotaurine (HT) to faba bean (*Vicia faba*) reverses ABA-induced effects. The phenotypes of ABA-insensitive mutants (*abi1*, *abi3*) suggest that H_2_S is a key player in ABA-mediated stomatal regulation via ion channels [29]. H_2_S binding to ABI1 promotes ABA-induced stomatal closure in *Arabidopsis*, an effect that is lost in the *abi1* mutant [63]. H_2_S may represent a novel component of the ABA-dependent defense signaling network that contributes to stomatal closure [64, 65]. Under drought conditions, H_2_S suppresses the expression of *Protein Phosphatase Type 2C* (*PP2C*) genes by upregulating *Pyrabatin Resistance/Pyrabatin Resistance-Like* (*PYR/PYL*) genes in wheat [66]. Moreover, H_2_S regulates stomatal movement through the persulfidation of SnRK2.6, an SNF1-related serine/threonine-protein kinase that is a core component of the ABA signaling pathway [45]. The guard cell-localized desulfhydrase DES1 also contributes to ABA-induced stomatal closure [52]. Collectively, H_2_S and ABA signaling pathways coregulate stomatal closure, most likely through the persulfidation of key signaling components [67].

Auxin, the first-discovered plant hormone, regulates stem elongation, inhibits lateral bud growth, and promotes rooting. H_2_S stimulates adventitious root formation, whereas an auxin (indole-3-acetic acid, IAA) transport inhibitor reduces H_2_S-induced root development [68]. The synthetic auxin 1-naphthaleneacetic acid (NAA) enhances lateral root formation in tomatoes by increasing H_2_S production. H_2_S also modulates the expression of genes encoding actin-binding proteins and auxin receptors, thereby influencing root growth [69]. Furthermore, H_2_S restricts primary root elongation by affecting polar auxin transport and IAA distribution [70]. Thus, the balance between H_2_S and auxin plays a crucial role in regulating root morphogenesis [71].

Gibberellic acids (GAs) control various developmental processes, including cell elongation, seed germination, flowering, and fruit set. In wheat seeds, both H_2_S and GA, individually or in combination, improve germination by increasing β-amylase activity in a time-dependent manner [72]. Additionally, H_2_S counteracts GA-induced programmed cell death [73]. It may also function as a downstream signaling molecule in the GA pathway in tomatoes to alleviate boron stress [74]. These findings suggest that H_2_S modulates seed germination under both normal and stress conditions through interactions with GAs.

Ethylene (Eth) promotes fruit ripening, leaf senescence, and the formation of adventitious roots and root hairs. It also plays a role in breaking seed and bud dormancy. Exogenous Eth induces H_2_S production in guard cells, leading to stomatal closure by activating LCD and DCD, an effect reversed by H_2_S synthesis inhibitors in *Arabidopsis* and *V. faba* [75, 76]. H_2_S also delays senescence in leafy vegetables by suppressing Eth production [77] and downregulates the expression of Eth synthetase and pectate lyase genes in banana, thereby slowing fruit ripening [78]. Additionally, H_2_S inhibits Eth production by reducing the activity of cell wall-modifying enzymes [26].

Salicylic acid (SA) plays a vital role in plant responses to both biotic and abiotic stresses. In maize seedlings, root irrigation with SA activates LCD, increases endogenous H_2_S levels, and enhances heat tolerance, whereas treatment with the H_2_S biosynthesis inhibitor DL-propargylglycine (PAG), or the H_2_S scavenger HT reduces H_2_S concentration [79]. SA also enhances LCD activity and H_2_S production under Cd-contaminated conditions, improving Cd stress tolerance [80]. In cucumber seedlings, H_2_S does not affect endogenous SA levels under normal conditions, but SA stimulates *LCD* and *DCD* gene expression, leading to H_2_S accumulation [81].

Recent studies on jasmonic acid (JA) have increasingly explored its role in plant responses to abiotic stress. In faba bean, exogenous JA enhances LCD and DCD activity, raises endogenous H_2_S levels, and induces stomatal closure [82]. JA also triggers Eth-mediated organ abscission [83], while H_2_S counteracts this by suppressing cellulase and polygalacturonase (PG) activities in tomatoes [26]. Additionally, although JA inhibits stomatal development, H_2_S reverses this inhibition in *Arabidopsis* [55].

Melatonin (MEL), a recently characterized phytohormone, regulates various physiological processes and stress responses. In chili plants, foliar MEL application alleviates iron deficiency and salt-induced damage by increasing endogenous H_2_S levels, an effect that is reversed by the H_2_S scavenger HT [84]. Furthermore, H_2_S and MEL jointly mediate crosstalk involving NO and Eth, thereby regulating metabolic pathways linked to fruit ripening [85]. More directly, H_2_S facilitates MEL-induced stomatal closure by modulating K^+^ channels in *Arabidopsis* [86].

### H_2_S and ROS

H_2_S and ROS function synergistically to regulate oxidative stress responses during plant growth, development, and stress adaptation. Pretreating seeds of the biodiesel plant *Jatropha curcas* with H_2_O_2_ enhances germination by increasing LCD enzyme activity, an effect reversed by aminooxy acetic acid, an inhibitor of H_2_S biosynthesis [87]. Conversely, H_2_S promotes H_2_O_2_ production by regulating RBOH activity, improving Na^+^/H^+^ transporter expression, and enhancing salt tolerance [88]. H_2_S also supports ROS scavenging by modulating antioxidant enzyme activity and maintaining redox balance [8], as well as increasing the biosynthesis of the nonenzymatic ROS scavenger glutathione [89]. Additionally, H_2_S inhibits excessive ROS accumulation and thereby delays postharvest senescence in grapes [90]. Mechanistically, H_2_S promotes RESPIRATORY OXIDASE HOMOLOG D (RBOHD) persulfidation at cysteine (Cys)^825^ and Cys^890^, enhancing ROS production associated with ABA-induced stomatal closure [91]. Both H_2_S and ROS mediate post-translational modifications (PTMs) of proteins at Cys residues. Cys thiol oxidation to sulfenic acid (RSOH), referred to as S-sulfenylation, enables H_2_S to form persulfides (RSSH). These persulfides can either revert to thiols via thioredoxin or react with ROS to form adducts (RSSO_3_H), which thioredoxin subsequently cleaves to regenerate thiols and sulfites. RSOH thus serves as a critical intermediate in both S-sulfenylation and persulfidation [92].

### H_2_S and NO

In plants, the interplay between H_2_S and NO is primarily evident during stomatal regulation. H_2_S induces stomatal closure, mirroring the role of NO [61]. H_2_S enhances NO production in guard cells and stimulates NO-mediated 8-mercapto-cyclic GMP (guanosine monophosphate) synthesis, resulting in stomatal closure [93]. Additionally, NO elevates H_2_S levels, thereby boosting plant tolerance to heat stress [94]. This synergy between H_2_S and NO is facilitated by upregulated nitrate reductase and glyoxalase I/II activities, along with H_2_S-mediated inhibition of *S*-nitrosoglutathione reductase activity [95, 96]. NO pretreatment enhances ROS detoxification under drought stress in alfalfa (*Medicago sativa* L.) and *Arabidopsis*, which aligns with H_2_S effects during drought response [97–99], with both gases functioning through PTM of Cys residues [92]. H_2_S and NO modify protein cysteine residues via persulfidation and S-nitrosylation, respectively [94, 100, 101], altering protein conformations and regulating enzyme activity to facilitate signal conversion and transmission [102].

### H_2_S and CO

Heme oxygenase (HO-1) catalyzes CO production in plants; however, the interaction between H_2_S and CO signaling remains less explored than that between H_2_S and other signaling molecules. H_2_S enhances *HO-1* expression, promoting adventitious root formation in cucumber, suggesting that H_2_S may act upstream of CO in root morphogenesis control [103]. Additionally, H_2_S modulates *HO-1* expression and CO production, alleviating GA-induced programmed cell death in wheat [73].

### H_2_S and Ca^2+^/calmodulin

Ca^2+^, an essential ion in numerous physiological processes in plants, plays a central role in various cellular functions. H_2_S induces stomatal closure and improves drought tolerance by regulating Ca^2+^ levels in guard cells [104]. Moreover, Ca^2+^/calmodulin (CaM) promotes endogenous H_2_S accumulation, enhancing heat tolerance in tobacco cell suspensions [105]. Notably, the bZIP transcription factor TGA3, which belongs to the ‘TGACG’-binding factor (TGA) family, enhances H_2_S production under hexavalent chromium (Cr^6+^) stress by binding to the *LCD* promoter, a process positively regulated by Ca^2+^ and the calmodulin isoform CaM2 [36]. This highlights Ca^2+^/CaM as an upstream regulator of H_2_S biosynthesis, offering valuable insights into the broader H_2_S signaling network.

### H_2_S and Cys

Although Cys is a nonessential amino acid, it serves as a crucial reducing agent and the primary organic donor of reduced sulfur in plants—both vital for sulfur metabolism. Notably, Cys is the main substrate for endogenous H_2_S production, and the pyridoxal-*O*-acetylserine(thiol)lyase (OASTL) family regulates the reversible reactions between H_2_S and Cys [106]. This points to a cyclical relationship between H_2_S and Cys in nature, potentially forming a regulatory loop during plant responses to heavy metal stress. The H_2_S–Cys pathway plays a significant role in the cellular mechanisms that confer Cr^6+^ stress tolerance [107].

### H_2_S and microRNAs

H_2_S also interacts with microRNAs (miRNAs), modulating the expression of miRNA-coding genes to enhance drought tolerance in *Arabidopsis* [108]. However, the precise relationship between H_2_S and miRNAs remains underexplored and merits further investigation.

### The main functional mechanisms of H_2_S

Recent studies have shed light on the functional mechanisms of H_2_S, particularly protein persulfidation, where Cys residues are oxidized to RSSH at the post-translational level [109–112]. This modification affects the structure, localization, and activity of target proteins, thereby influencing various biological processes [113–115]. Protein persulfidation is now widely regarded as a central mechanism of H_2_S signaling. For example, H_2_S inhibits 1-aminocyclopropane-1-carboxylic acid (ACC) oxidase (ACO), the final enzyme in the ethylene biosynthesis pathway via persulfidation, regulating root hair elongation [116]. It also enhances the persulfidation of flowering locus C (FLC) in Chinese cabbage to modulate flowering [19]. Additionally, this modification is critical for plant stress responses [18], as it regulates autophagy by modifying the *ATG4A*-encoded protein and helps maintain ROS homeostasis under stress conditions [117, 118]. However, the detailed molecular mechanism underlying the conversion of cysteine residues (RSH) to persulfides (RSSH) remains unclear and requires further investigation.

**Figure 4:**
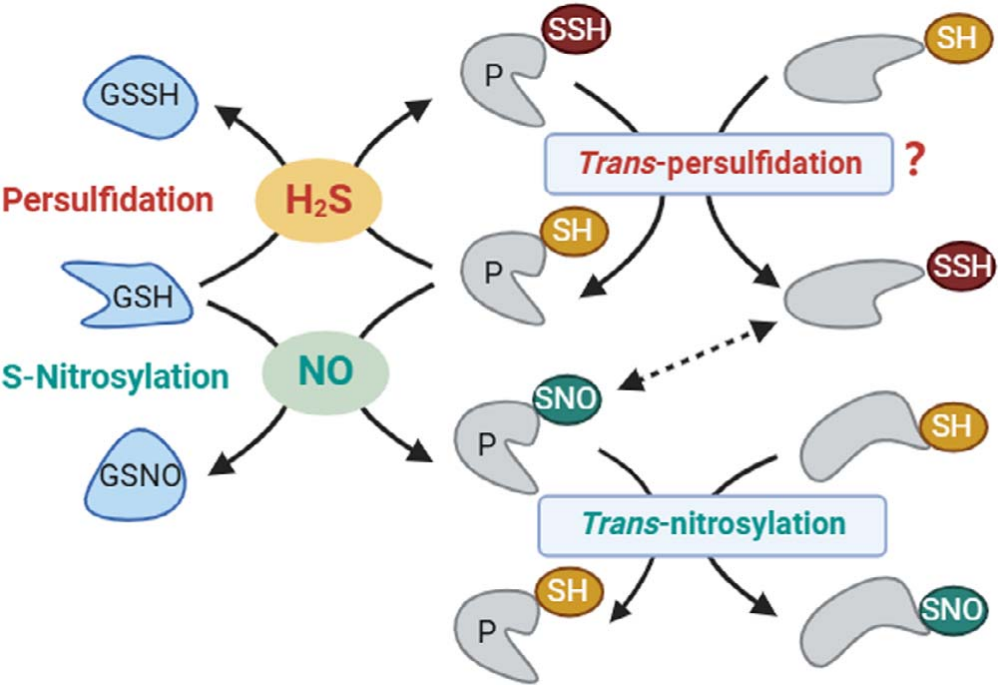
The mechanism and relationship between H_2_S and NO function [102]. GSH, reduced glutathione; GSSH, glutathione persulfide; GSNO, nitrosoglutathione; P, protein; SH, thiol group; SSH, sulfhydryl group; SNO, nitroso group.

H_2_S and NO, both gaseous signaling molecules, share similar mechanisms involving redox-based PTMs—S-nitrosylation and persulfidation—that often target the same Cys residues. In mammals, 36% of the proteome exhibits overlap between these modifications [119], and 639 proteins undergo both modifications in *Arabidopsis* [101]. However, their effects differ: S-nitrosylation inhibits, whereas persulfidation enhances the activity of glyceraldehyde 3-phosphate dehydrogenase (GAPDH) [110, 120]. Persulfidation of Cys^131^ and Cys^137^ activates SnRK2.6/OPEN STOMATA 1 (OST1), while S-nitrosylation at Cys137 conversely suppresses its activity [100]. Moreover, phosphorylation of SnRK2.6 further enhances persulfidation, thereby modifying its function [45, 121]. The crosstalk between persulfidation and other PTMs is complex and may involve synergistic or antagonistic interactions, highlighting the need for further investigation (Fig. 4).

Developing reliable assays to detect PTMs has been challenging. Over the past decade, several validated methods have been refined to study persulfidation in H_2_S signaling target proteins. Initially, a modified biotin-switch technique was employed: methyl methanesulfonate (MMTS) blocked Cys thiols (–SH), and biotin-HPDP was used to label and enrich –SSH groups [113]. Subsequently, fluorescent reagents such as maleimide (Mal) were utilized to label both –SSH and –SH, forming –SS-Mal and –S-Mal adducts, respectively [122, 123]. Alkylating agents like iodoacetic acid have also been applied to block sulfhydryl and persulfide groups [124]. The tag–switch method was later introduced to quantify persulfidation levels [125], and a direct proteomic assay for persulfidation detection has since been developed [126]. Despite these advances, the development of highly specific, sensitive, and user-friendly detection tools for PTMs remains a key challenge for future research.

## Research progress on the function of H_2_S in horticulture

The physiological functions and mechanisms of H_2_S in the model plant *Arabidopsis*, a close relative of the horticulturally important *Brassica* genus, have been extensively studied. As a result, researchers have increasingly turned their attention to exploring the potential applications of H_2_S in horticulture (Fig. S1).

### Function of H_2_S in vegetables

The regulation of H_2_S signaling in vegetables has been investigated, particularly in members of the Brassicaceae, Solanaceae, Cucurbitaceae, Amaryllidaceae, Apiaceae, and Amaranthaceae families (Table 1). Most research relies on exogenous H_2_S application, except in tomatoes, which benefit from a well-established genetic transformation system [127, 128]. For vegetables, exogenous H_2_S is typically applied at concentrations ranging from 0.1 to 1 mM [19, 129–131]—about 10 times higher than in *Arabidopsis*, likely due to species-specific differences in endogenous H_2_S levels and overall plant biomass [132]. Many studies examine how H_2_S treatment mitigates heavy metal stress [131, 133–136], including aluminum [137], lead [138, 139], nickel [140], copper [141], and other abiotic stress such as salinity [142–144], drought [17, 145], and chilling [146, 147]. In addition, the role of H_2_S in vegetable growth and development [19, 27, 116, 128, 129, 148–153] and in postharvest processes [22, 77, 154, 155] have been actively explored.

**Table 1:** Application of H_2_S to vegetables

**Family**	**Species**	**Development/stress**	**Growth stage**	**H**_**2**_ **S dose**	**Mechanism**
Brassicaceae	*Brassica chinensis*	Lead stress	5-leaf stage	100, 200 μM	Photosynthesis
		Cd stress	5-leaf stage	100, 200 μM	Antioxidant system
		Cd stress	4-day-old seedlings	50 μM	Demethylation, cell wall
		Cd stress	Radicle 1.0 cm	0.1–0.5 mM	Antioxidant system
		Cd stress	20-day-old seedlings	1.5 mM	Antioxidant system, IAA
		Salinity stress	3-day-old seedlings	5 mM	Ionic homeostasis
		Aluminum stress	5-day-old seedlings	20 μM	Antioxidant system
	*Brassica pekinensis*	Sulfate nutrients	10-day-old seedlings	0.2 μM	Sulfate transporters, minerals
		Flowering	7-day-old seedlings	100 μM	BrFLC, persulfidation
		Drought stress	4-wk-old seedlings	100 μM	RBCL, persulfidation, ion channels
	*Brassica oleracea*	Postharvest senescence	Fresh broccoli head	2.4 mM	Antioxidant system
		Yellowing	Fresh broccoli head	0.8 mM	Chlorophyll degradation, energy metabolism
		Salinity stress	45-day-old plants	0.1, 0.2 mM	Antioxidant system, photosynthesis
	*Brassica rapa*	Storage and aging	Pakchoi	50, 100 μM	Antioxidant system, Eth
		Postharvest storage	Pakchoi	250 μl/L	Antioxidant system, PAG
		Cd stress	Cotyledons	0.3 mM	Antioxidant system
	*B. oleracea*	Regulation of APR	10-day-old seedlings	0–1.2 μM	Sulfate uptake
		Antioxidant capacity	43-day-old plants	0.5, 1 mM	Antioxidant system
	*Eruca sativa*	Dehydration	1-wk-old seedlings	2 mM	Cysteine biosynthesis
	*Brassica juncea*	Cd stress	15-day-old seedlings	100 μM	Antioxidant system, photosynthesis
Solanaceae	*Solanum lycopersicum*	Lateral root formation	3-day-old seedlings	1 mM	DES, CDKA1, CYCA2, hormone
		Nitrate stress	3-wk-old seedlings	100 μM	Antioxidant system
		Ethylene synthesis	2-wk-old seedlings	200 μM	Stomatal closure PTM of ACO
		Fruit ripening	Fruits	0.9 mM	Ethylene
		Lateral root formation	3-day-old seedlings	1 mM	DES
		Postharvest	Ripe fruits	0.9 mM	Ethylene, photosynthesis
		Fruit ripening	Fruits	0.6 mM	SlLCD1, sulfur metabolism
		Cr stress	30-day-old seedlings	50 μM	GSH
		Salinity stress	2-leaf stage	40 μM	Antioxidant system
		Fruit ripening	Green fruits Flowering	0.6 mM	Ethylene, methionine synthase
		Petiole abscission	6-wk-old seedlings	50 μM	Antioxidant system, ethylene
		Fruit ripening	Fruits	50 μM	Antioxidant system, ethylene
		Fruit ripening	Fruits	0.9 mM	Ubiquitination, persulfidation
	*Capsicum annuum*	Chilling stress	6-leaf stage	1 mM	Photosynthesis
		Lead stress	1-wk-old seedlings	0.2 mM	AsA-GSH
		Chilling stress	6-leaf stage	1 mM	Antioxidant system, AsA-GSH
	*Capsicum annuum* L.*,* cv. Melchor	Fruit ripening	Fruits	5 ppm NO	NO
	*Solanum melongena*	Salinity stress	2-leaf stage	0–100 μM	Antioxidant system, ABA
Cucurbitaceae	*Cucumis sativus*	Nitrate stress	3-leaf stage	100 μM	Antioxidant system, MAPK/NO
		Chilling stress	3-leaf stage	50 μM	CuC synthase, sulfur metabolism
		Chilling stress	2-leaf stage	0.5–2.5 mM	Photosynthesis, carbon metabolism
		Salt tolerance	2-wk-old seedlings	200 μM HT	Antioxidant system, photosynthesis, NO/MAPK
		Cu stress	10-day-old seedlings	100 μM	Antioxidant system, proline metabolism
		Salinity stress	2-leaf stage	200 μM	Photosynthesis, AsA-GSH, SOS, MAPK
	*Cucurbita pepo*	Seed germination	10-day-old seedlings	100 μM	AsA-GSH, Redox homeostasis phytochelatins
Amaryllidaceae	*Allium sativum*	H₂S release activity	Clove		Sulfur metabolism
		Vascular regulation	Clove	40 μM	Sulfur metabolism
Apiaceae	*Daucus carota*	Surface whitening	Root slices	0.4 mM	Antioxidant system
Amaranthaceae	*Spinacia oleracea*	Cd stress	Cotyledons	100 μM	Antioxidant system

Although mode-of-action studies on H_2_S in vegetables remain less systematic, they emphasize its role in antioxidant defense. For instance, H_2_S stimulates growth by activating antioxidant systems in kale [156] and, in combination with SA, alleviates Cd stress by enhancing antioxidant activity, nutrient uptake, photosynthesis, and biomass accumulation in mustard [157]. More detailed findings indicate that H_2_S promotes flowering through FLC persulfidation in Chinese cabbage [19] and modulates tomato fruit ripening via target protein persulfidation [155]. Given widespread heavy metals contamination in agricultural soils, further basic research is critical to fully harness H_2_S applications for enhancing metal tolerance and improving crop yields.

### Function of H_2_S in fruits

H_2_S plays a significant role in the postharvest preservation of fruit, including those from the Rosaceae, Actinidiaceae, Musaceae, Moraceae, and Ericaceae families. Studies have examined how H_2_S regulates ripening and senescence, as well as its application in low-temperature storage. Research on strawberry [158–164], pear [42, 165], peach [166–168], apple [169, 170], kiwi [171, 172], banana [173, 174], mulberry [175], and blueberry [176] under cold stress reveals that H_2_S exhibits a dose-dependent beneficial effect. Fruits treated with H_2_S show reduced decay, enhanced firmness, and altered respiration rates and PG activity levels [158, 163, 177]. Applied at concentrations of 0.1–2 mM, H_2_S boosts antioxidant activity, reduces ROS levels and lipoxygenase activity (involved in JA biosynthesis), and preserves reducing sugars, soluble proteins, free amino acids, and endogenous H_2_S, thereby maintaining cellular integrity and prolonging shelf life.

Key fruit preservation strategies include shelf life extension, antioxidant application, the use of preservatives, and cold storage [168, 178–181]. H_2_S effectively delays softening, suppresses respiration, reduces decay, modifies water transport and cell wall metabolism [158, 163, 177], and supports mineral nutrient balance along with antioxidant enzyme activity [158, 160, 163, 169]. These attributes suggest that H_2_S treatment may be an effective postharvest strategy for maintaining the quality of horticultural products during storage and transport (Table 2).

**Table 2:** Application of H_2_S to fruits

**Family**	**Species**	**Development /stress**	**Growth stage**	**H**_**2**_ **S dose**	**Mechanism**
Rosaceae	*Fragaria × ananassa*	Oxidative damage	Fruits	0.8 mM	Antioxidant system
		Heat stress	187-day-old	100 μM	Heat shock defense-related pathways
		Iron deficiency stress	2-wk-old	0.2 mM	Antioxidant system, mineral balanced
		Heat stress	3-leaf stage	1.25 mM	Antioxidant system
		Cd stress, hot stress	2-wk-old	0.2 mM	Antioxidant system, NO
		Alkaline stress	3-month-old	0.2 mM	Antioxidant system
		Postharvest	Fruits	0.2 mM	Pectinase
	*Pyrus pyrifolia*	Oxidative damage	Fruits	0.5 mM	Antioxidant system
		Persulfidation	Fruits	0.9 mM	Anthocyanins
	*Prunus persica*	Postharvest	Fruits	20 μM	NO, ethylene
		Postharvest	Fruits	10 μM	Antioxidant system
		Low-temperature stress	Fruits	20 μM	NAD/NADP
	*Malus pumila*	Postharvest	Fruits	0.7 mM	Antioxidant system, energy metabolism
		Salt stress	2-wk-old	0.5 mM	Carbon metabolism
		Postharvest	Fruits	0.7 mM	Lipid metabolism
Macacaillaceae	*Actinidia chinensis Planch*	Postharvest	Fruits	20, 40 mM	MDA, cell membrane
		Postharvest	Fruits	20 μM	JA
Musaceae	*Musa nana*	Postharvest	Fruits	1 mM	Photosynthesis, ethylene
		Low-temperature stress	Fruits	2 mM	Photosynthesis, antioxidant system
Moraceae	*Morus indica*	Postharvest	Fruits	0.8 mM	Respiration, antioxidant system
Ericaceae	*Vaccinium spp.*	Low-temperature stress	1-yr-old	0.5 mM	Stomatal movement, photosynthesis

### Function of H_2_S in other species

Research on H_2_S signaling has significantly progressed across various plant families. In grasses (Poaceae family), such as tall fescue [180] and Bermuda grass [182], H_2_S alleviates abiotic stress by regulating antioxidants and genes related to chlorophyll synthesis. In the Asteraceae family, including safflower [183], *Artemisia annua* [184], and artichoke [185], H_2_S boosts secondary metabolite production, antioxidant capacity, and biomass retention under salt and heavy metal stress. In Rosaceae species, H_2_S alters fresh weight and ROS levels while increasing soluble sugars, proteins, anthocyanins, carotenoids, and antioxidant enzyme activities [186, 187]. In legumes (Fabaceae family), such as Trigonella [188] and alfalfa [189–191], H_2_S helps regulate osmotic balance, antioxidant systems, and phenolic and flavonoid content. In trees from the Salicaceae [96, 192], Euphorbiaceae [87], and Juglandaceae [193] families, H_2_S modulates photosynthesis, antioxidant responses, NO synthesis, carbohydrate metabolism, and redox homeostasis. H_2_S also contributes to tolerance against heavy metals [184, 188], cold [189], and salt stress [11, 193]. Additionally, H_2_S has shown potential in economically significant crops like tobacco [194–196] and even in prokaryotes such as cyanobacteria [197]. Due to species-specific variations in growth stages and tissue types, the applied concentrations of H_2_S differ considerably (Table 3).

**Table 3:** Application of H_2_S to other species

**Family**	**Species**	**Development stage/stress**	**Growth stage**	**H**_**2**_ **S dose**	**Mechanism**
Asteraceae	*Carthamus tinctorius*	Oxidative damage	3-yr-old	0.5, 1 mM	Secondary metabolites, antioxidant system
		Lead stress	21-day-old	100 μM	Ascorbate-glutathione cycle
	*Artemisia annua*	Cu stress	4-wk-old	200 μM	Lipid peroxidation, antioxidant system
	*Cynara scolymus*	Saline, aniline stress	45-day-old	200 μM	Permeable substance
Solanaceae	*Nicotiana tabacum*	Salt stress	3-leaf stage		Aantioxidant system
		Cd stress	14-day-old	0.9 mM	Photosynthesis
		Seeds	14-day-old	0–8 mM	Antioxidant system
Rosaceae	*Malus hupehensis*	Saline, alkaline stress	6-leaf stage	0.5 mM	Energy maintenance, antioxidant system
	*Rosa hybrida*	Oxidative damage	Petal	50 μM	Pigmentation, antioxidant system
Poaceae	*Festuca arundinacea Schreb*	Low light stress	28-day-old	500 μM	Photosynthesis
	*Cynodon dactylon*	Cd stress	21-day-old	500 mM	Antioxidant system
Salicaceae	*Populus trichocarpa*	Cd stress	10-day-old	50–100 mM	Antioxidant system
	*Populus euphratica Oliv*	Heat stress	3-wk-old	50 μM	NO, GSNOR
Juglandaceae	*Cyclocarya paliurus*	Salt stress	8-month-old	0.5 mM	Antioxidant system
		Salt stress	6-month-old	0.5 mM	Photosynthesis, antioxidant system, NO
Rhizophoraceae	*Kandelia obovata*	Salt stress	3rd pair of blades	200 μM	Photosynthesis
		Salt stress	Trees	0.01–5 mM	Carbohydrate metabolism, redox homeostasis
Euphorbiaceae	*Jatropha curcas*	Seeds	7-day-old	0.5, 1 mM	Antioxidant system, LCD
Cyanobacteriaceae	*Cyanobacteria*	Darkness, hypoxic stress		150 μM	Photosynthesis

H_2_S signaling studies extend to food crops, particularly members of the Poaceae (wheat, rice, foxtail millet, barley, and corn) and Fabaceae (soybean, mung bean, pea, and faba bean), underscoring the role of H_2_S in stress alleviation by enhancing photosynthesis, sugar metabolism, and antioxidant system activity (Table S1). H_2_S also interacts with rhizobacteria, Ca^2+^, and NO to modulate plant functions. Limited research on other crops, such as sesame and sweet potato, suggests that H_2_S improves mineral balance and tuber development, respectively [54, 198–215]. Overall, H_2_S plays a crucial role in crop cultivation, with potential applications for improving stress responses and productivity through advanced agricultural strategies.

## H_2_S signal production in plants

**Figure 5:**
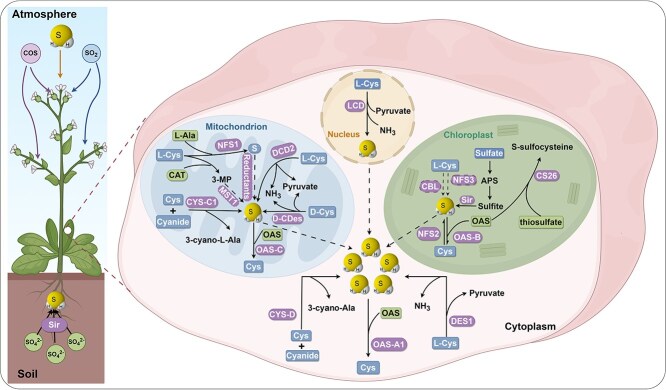
The uptake and biosynthesis of H_2_S in *Arabidopsis.* APS, adenosine phosphosulfate; CAT, cysteine aminotransferase; CBL, cystathionine beta-lyase; COS, carbonyl sulfide; CS26, cysteine synthase 26; Cys, cysteine; CYS-C1, cysteine synthase-C1; DCD, D-cysteine desulfhydrase; DES1, desulfhydrase 1; MST, mercaptopyruvate transferase; 3-MP, 3-mercaptopyruvate; NFS1, nitrogen fixation S 1; OAS, *O*-acetylserine; Sir, sulfite reductase.

In higher plants, H_2_S originates from three main sources (Fig. 5) [106]. Cys degradation, catalyzed by L/DCD, is the primary source. The other two key sources include sulfite reduction to H_2_S by sulfite reductase with sulfate and sulfite absorbed through the roots, wherein SO_3_^2−^ may also be converted to Cys [216], and atmospheric absorption through the leaves, including carbonyl sulfide (COS) hydrolyzed by carbonic anhydrase and SO_2_ hydrolyzed to sulfite [217]. In *Arabidopsis*, H_2_S production involves enzymes such as cysteine desulfhydrase (CDes), nitrogen fixation S (NFS/Nifs), OASTLs containing β-cyalanine synthase (CYS), which are localized in the cytoplasm, chloroplasts, and mitochondria, respectively. Collectively, these pathways sustain trace H_2_S concentrations across diverse plant species, tissues, and developmental stages (Table S2) [132].

With a deeper understanding of H_2_S production in plants, genetic engineering has enabled the development of knockout mutants and overexpression lines targeting genes involved in H_2_S biosynthesis and utilization, facilitating detailed exploration of the physiological roles of H_2_S in plants. Specifically, variants such as OE-*LCD/lcd* [14, 29, 34, 47, 53], OE-*DES1/des1* [47, 75, 109], *oastl* [218, 219], OE-*DCD*/*dcd* [14, 35, 53], and *oasa1* [14] have been developed and studied, providing vital genetic resources for H_2_S functional research.

## Future prospects

### Receptor identification is necessary for H_2_S signaling elucidation

Whether gasotransmitters rely on traditional receptors for signal transmission has been a matter of debate [1–3]. NO, the most well-studied gasotransmitter, binds to soluble guanylate cyclase (sGC) via heme iron (Fe^2+^), triggering cGMP production and downstream activation of PKG and phosphodiesterases—effectively making sGC its receptor. NO also regulates protein function through S-nitrosylation of cysteine residues (e.g. in ion channels, MAP kinases) [94]. In contrast, H_2_S has not yet been clearly confirmed to have specific membrane or intracellular receptors. Its signaling primarily involves cysteine residues of target proteins via persulfidation, thereby modulating protein conformation and function. Proposed H_2_S targets include H_2_S-generating enzymes, autophagy pathways, ROS systems, hormone signaling, actin/glucose metabolism, though research remains fragmented. The diversity and dynamism of target proteins may also be the primary reason for the current functional diversity of H_2_S. Additionally, some scholars believe that the primary function of H_2_S is to regulate cellular redox balance [18]. Further research may reveal more specific traditional receptor mechanisms, opening up new avenues of exploration.

### Anion channels are potential transporters for HS^−^ homeostasis

While the receptor-dependent signaling of H_2_S remains controversial, its unique membrane permeability and ionic regulation mechanisms provide complementary insights into its biological actions. Under physiological conditions, >80% of H_2_S exists as HS^−^ due to its pKa of 6.8, with smaller proportions as H_2_S or S^2−^ [2, 220]. The pH gradient between intracellular (neutral) and extracellular (slightly acidic) environments could lead to a 10- to 100-fold accumulation of intracellular HS^−^ if not properly regulated, underscoring the critical need for efficient export mechanisms. To date, researchers have identified several HS^−^ transporters, including bacterial hydrosulfide ion channels (HSC) and human erythrocyte anion exchangers (AE1) [221, 222]. Inspired by these natural systems, scientists have also developed synthetic HS^−^ receptors using reversible noncovalent interactions [221, 223]. However, the transport mechanisms for H_2_S/HS^−^ in plants remain completely unknown. Key questions include how H_2_S/HS^−^ are transported in plant cells, whether specific HS^−^ channels exist, and if so, whether they share pathways with other anions. Addressing these questions will broaden our understanding of H_2_S metabolism in diverse organisms and support further exploration of its physiological roles, molecular recognition mechanisms, and protein targets.

### Splicing factors are potential targets for H_2_S signaling

The well-characterized mechanism of H_2_S signaling involves the persulfidation of reactive cysteine residues in target proteins. Proteomic studies indicate that at least 5% of *Arabidopsis* proteins may undergo persulfidation, regulating key primary metabolic pathways such as the Calvin cycle, glycolysis, and the tricarboxylic acid (Krebs) cycle [109]. Recent findings reveal that H_2_S also influences the alternative splicing of flowering-related genes via the persulfidation of the splicing factor AtU2AF65a [20] and BraATO2 [224], resulting in premature flowering. Furthermore, it was also discovered that H_2_S modulates *BrSDH1-1* alternative splicing to induce stomatal closure in Chinese cabbage [225]. These discoveries deepen our understanding of H_2_S signaling at the posttranslational level. Alternative splicing is widespread in plants, enhancing genetic diversity and playing essential roles in growth, development, and stress responses [226]. Proteomic analyses suggest that H_2_S modulates splicing factors, which subsequently affect the transcription of downstream genes [109, 110]. Additional studies are required to determine the significance of splicing factors as H_2_S signaling targets. Such research could yield fresh insights into H_2_S signal transduction and its potential applications in agriculture.

### Retarder preparation is necessary for H_2_S applications

The dual nature of H_2_S being toxic at high concentrations but a vital signaling molecule at low levels has complicated efforts to determine its precise physiological concentrations and to develop effective exogenous application methods. Common donors such as NaHS and Na_2_S release H_2_S rapidly in water. This results in unstable effects, short action durations, increased costs and toxicity risks when applied in large-scale agriculture [227]. To overcome these limitations, slow-release H_2_S agents are necessary. GYY4137, a water-soluble molecule, enables stable and controllable H_2_S delivery via intracellular redox reactions lasting from hours to days, making it suitable for studies involving chronic conditions [228]. Recent approaches involve COS release via thiocarbamates, which generate H_2_S and carbon dioxide (CO_2_) in the presence of carbonic anhydrase, though separating their respective effects remains a challenge [229]. However, both GYY4137 and COS are currently too costly for widespread agricultural use. Therefore, developing low-cost, environmentally friendly slow-release H_2_S agents is essential. Such innovations could enhance the efficiency of H_2_S utilization, streamline agricultural practices, and improve crop yield and quality, making them a priority for future research.

The development of controlled-release agents is critical for the broader application of H_2_S in agriculture. Its stress-alleviating properties can be synergized with nanomaterials to create effective ‘biostimulants’ aimed at boosting crop productivity. H_2_S modulates target genes at both transcriptional and post-translational levels, thereby influencing biological processes and agronomic traits such as yield and quality, while also aiding in the identification of relevant protein targets. With advances in gene editing and growing demand for high-quality produce, non-GMO CRISPR/Cas9 technology offers potential for fine-tuning the regulation of key genes, significantly improving agricultural output.

### Gasotransmitter origins are clues for functional insights

H_2_S is the third gas signaling molecule identified after NO and CO. Additionally, ammonia (NH_3_), methane (CH_4_), and hydrogen (H_2_) have gradually exhibited common characteristics of gas signaling molecules [1–3]. These gas molecules perform irreplaceable positive physiological functions in organisms at physiological concentrations. This might be puzzling: why are they all considered ‘toxic gases’ in our traditional understanding? The Earth’s primitive atmosphere contained various gases such as H_2_, NH_3_, CH_4_, H_2_S, CO, NO, CO_2_, helium (He), and sulfur dioxide (SO_2_), which significantly differs from current atmosphere primarily composed of nitrogen (N_2_) and oxygen (O_2_). Many components of the primitive atmosphere have been reported to possess specific physiological functions. This may suggest that with the changes in the components of the primitive atmosphere during the long process of biological adaptation and evolution, organisms have retained primitive traces of utilizing these gaseous components, which continue to play important physiological roles in modern species. Exploring the relationship between changes in atmospheric composition and the physiological functions of gas molecules within living organisms may hold important guiding significance for research in this field [2].

In summary, the perception of H_2_S has shifted from a malodorous gas to a critical gaseous signaling molecule in plants, where it helps maintain cellular homeostasis during growth, development, and environmental adaptation. Transitioning from ‘odor to order’ and ‘stone to jade’, H_2_S is well positioned to play a transformative role in future agricultural production.

## Supplementary Material

Web_Material_uhaf273
