# Supplementary material for: From odor to order: unveiling the crucial role of hydrogen sulfide in plant life
Source: Hortic Res. 2025 Oct 17;13(1):uhaf273. doi: 10.1093/hr/uhaf273 (PMC12881861; doi:10.1093/hr/uhaf273)
Supplement: Web_Material_uhaf273 [file web_material_uhaf273.zip › Supplementary materials.docx]

**From Odor to Order: Unveiling the Crucial Role of Hydrogen Sulfide in Plant Life**

Zhuping Jin

School of Life Science, Shanxi Key Laboratory for Research and Development of Regional Plants, Shanxi University, Taiyuan, Shanxi Province 030031, China

E-mail: jinzhuping@sxu.edu.cn


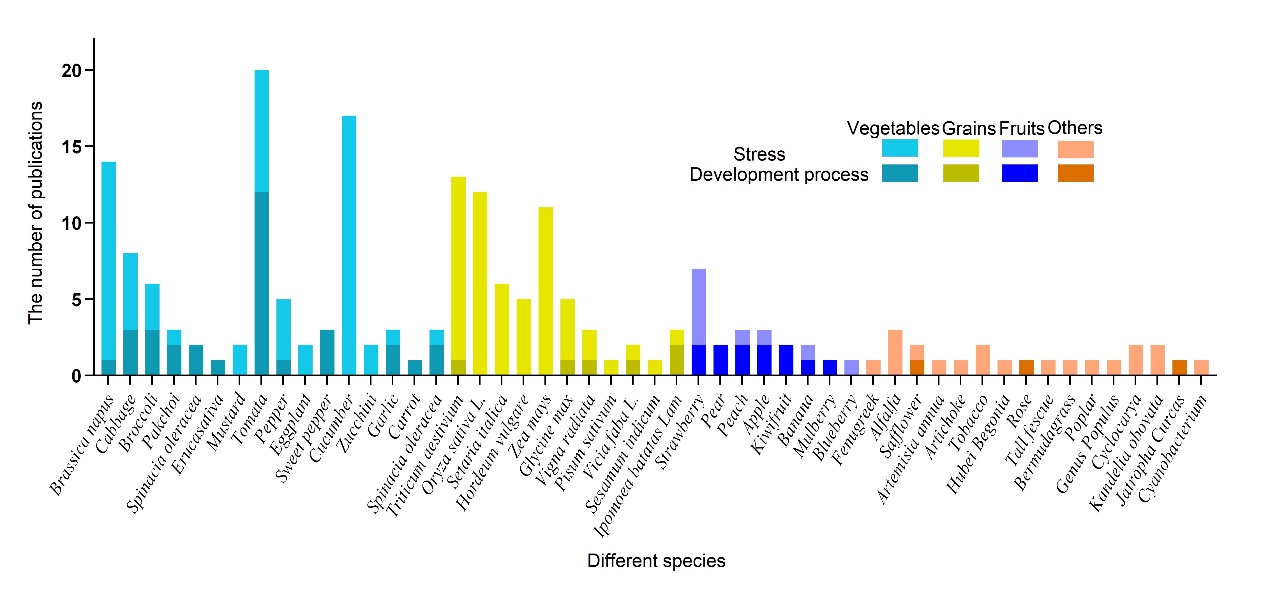


**Figure S1. The publications studied the application of H_2_S in crops.**

The green color family represents vegetables, yellow represents grains, blue represents fruits and orange represents others. The lighter shades of each color represent the development process, while the darker shades represent stress. The data are from Web of Science.

**Table S1.** Application of H_2_S to grains

| **Family** | **Species** | **Development /stress** | **Growth stage** | **H_2_S dose** | **Mechanism** |
| --- | --- | --- | --- | --- | --- |
| Poaceae | *Triticum aestivum* | Salt stress | 10-d-old | 200 μM | Antioxidant system |
|  |  | Salt stress | 14-d-old | 20 μM | Antioxidant system, ion homeostasis |
|  |  | Cd stress | 7-d-old | 50 μM | Photosynthesis, carbohydrate metabolism |
|  | *Oryza sativa* | Heat stress | Sprout | 100 μM HT | Antioxidant system, carbohydrate metabolism |
|  |  | Al stress | Sapling | 2 μM | Antioxidant system, Al content |
|  |  | Cr stress | 21-d-old | 15 μM | Sulfur assimilation, ascorbate-glutathione cycle |
|  | *Setaria italica* | Cd stress | 5-d-old | 50 μM | Antioxidant system |
|  |  | Drought stress | 10-d-old | 50 μM | DNA methylation |
|  | *Hordeum vulgare* | Drought and heat stress | 14-d-old | 100 μM | Antioxidant system, hormones |
|  |  | UV-B stress | 4-d-old | 1 mM | Antioxidant system, secondary metabolite synthesis |
|  | *Zea mays* | Cr stress | 3-d-old | 50 μM | Chromium fixation, redox homeostasis |
| Leguminosae | *Medicago sativa* | Salt stress | 5-d-old | 50-300 mM | Antioxidant system, K^+^ externalization |
|  |  | Cd stress | 4-d-old | 100 µM | CH_4_ |
|  |  | Low-temperature stress | 5-w-old | 500 µM | Antioxidant system, photosynthesis |
|  | *Trigonella foenum-graecum* | Cd stress | 14-d-old | 100-200 µM | Antioxidant system, phenols, flavonoids |
|  | *Glycine max* | Aging | Sapling | 100 μm | Chlorophyll content, nitrogen reuptake |
|  |  | Nitrogen stress | 32-d-old | 100 μM | Photosynthesis |
|  | *Vigna radiata* | Cr stress | 20-d-old | 100 μM | Antioxidant system |
|  |  | Salt stress | 10-d-old | 1 mM HT | Antioxidant system, ionic homeostasis |
|  | *Pisum sativum* | Arsenic stress | 15-d-old | 100 μM | NO, AsA-GSH cycle |
|  |  | Hypoxia stress | 6-d-old | 100 μM | Antioxidant system |
|  | *Vicia faba* | Arsenic stress | 7-d-old | 2 mM | Antioxidant system, cysteine |
| Pedaliaeeae | *Sesamum indicum* | Lead stress | 30-d-old | 200 μM | Antioxidant system, mineral homeostasis |
| Dioscoreaceae | *Ipomoea batatas* | Root organogenesis | 7-d-old | 200 μM | IAA, NO |

**Table S2.** Enzymes generating H_2_S in *Arabidopsis thaliana*

| **Family** | **Enzyme** | **Gene Locus** | **Enzyme Commission Number** | **Cellular Localization** | **Substrate** | **Remark** |
| --- | --- | --- | --- | --- | --- | --- |
| CDes | LCD | At3g62130 | EC 4.4.1.1 | Nucleus | L-Cystine | PLP dependent |
|  | DCD1 | At1g48420 | EC 4.4.1.15 | Mitochondria | D-Cysteine |  |
|  | DCD2 | At3g26115 |  | Mitochondria | Cysteine |  |
| NFS | NFS1 | At5g65720 | EC 2.8.1.7 | Mitochondria | Cysteine |  |
|  | NFS2 | At1g08490 |  | Chloroplast |  | EC 4.4.1.16 |
| OASTL | DES1 | At5g28030 | EC 2.5.1.47 | Cytosol | Cysteine | EC 4.4.1.28 |
|  | OAS-A1 | At4g14880 |  | Cytosol |  |  |
|  | OAS-A2 | At3g22460 |  | Cytosol |  | Pseudogene |
|  | OAS-B | At2g43750 |  | Chloroplast |  |  |
|  | OAS-C | At3g59760 |  | Mitochondria |  |  |
|  | CYS-C1 | At3g61440 |  | Mitochondria | Cysteine  Cyanide | EC 4.4.1.9 |
|  | CYS-D1 | At3g04940 |  | Cytosol |  |  |
|  | CYS-D2 | At5g28020 |  | Cytosol |  |  |
|  | CS26 | At3g03630 | EC 2.5.1.144 | Chloroplast | Thiosulfate | SCS |
| Uncertain | CBL | At3g57050 | EC 4.4.1.13 | Chloroplast (Predicted) | L-Cysteine | Novel |
|  |  | At5g26600 | EC 4.4.1.28 | Chloroplast | L-Cysteine | Novel |
|  | MST1 | At1g79230 | EC 2.8.1.1 | Mitochondria  (Predicted) | Sodium mercapto  pyruvate | Novel |
|  | MST2 | [At1g16460](http://www.arabidopsis.org/servlets/TairObject?type=gene&id=1000438583) |  | Cytosol  (Predicted) |  | Novel |
| OR | SiR | At5g04590 | EC 1.8.7.1 | 7 components | Sulfite |  |
| CA | α-CA  β-CA  γ-CA  γ-CAL | 8 Genes  6 Genes  3 Genes  2 Genes | EC 4.2.1.1 | Cytosol  Chloroplast  Mitochondria  Mitochondria | Carbonyl sulfide | ** |
|  | β-CA | 6 Genes |  | Chloroplast |  |  |
|  | γ-CA | 5 Genes |  | Mitochondria |  |  |

**Gene locus of CA family: αCA1, At3g52720; αCA2, At2g28210; αCA3, At5g04180; αCA4, At4g20990; αCA5, At1g08065; αCA6, At4g21000; αCA7, At1g08080; αCA8, At5g56330; βCA1, At3g01500; βCA2, At5g14740; βCA3, At1g23730; βCA4, At1g70410; βCA5, At4g33580; βCA6, At1g58180; γCA1, At1g19580; γCA2, At1g47260; γCA3, At5g66510; γCAL1, At5g63510; γCAL2, At3g48680.

**Table S3.** Abbreviations

| Abbr. | Full name | Abbr. | Full name |
| --- | --- | --- | --- |
| ABA | abscisic acid | Eth | ethylene |
| ABP | actin-binding protein | GA | gibberellin |
| ACS | ACC synthase | GR | glutathione reductase |
| ACO | ACC oxidase | GSH | glutathione |
| APS | adenosine phosphosulfate | GSSG | glutathione disulfide |
| APX | ascorbate peroxidase | GSSH | glutathione persulfide |
| ASA | acetylsalicylic acid | GSNO | nitrosoglutathione |
| Aux | auxin | H_2_O_2_ | hydrogen peroxide |
| CA | carbonic anhydrase | IAA | indole-3-acetic acid |
| CAL | carbonic anhydrase like | JA | jasmonic acid |
| CAS | β-cyanoalanine synthase | LCD | L-cysteine desulfhydrase |
| CAT | catalase | MEL | melatonin |
| CAT | cysteine aminotransferase | 3-MP | 3-mercaptopyruvate |
| CaM | calmodulin | MST1 | mercaptopyruvate transferase 1 |
| CBL | cystathionine beta-lyase | NADPH | nicotinamide adenine dinucleotide  phosphate |
| CDes | cysteine desulfhydrases | NFS | nitrogen fixation S |
| cGMP | cyclic guanosine monophosphate | NO | nitric oxide |
| CO | carbon monoxide | OASTL | *O*-acetylserine (thiol) lyase |
| COS | carbonyl sulfide | PAT | polar auxin transport |
| CS26 | cysteine synthase 26 | PLP | pyridoxal 5’-phosphate |
| CYS | cysteine synthase | PYR/PYL | pyrabactin resistance/pyr-like |
| Cys | cysteine | ROS | reactive oxygen species |
| DCD | D-cysteine desulfhydrase | SA | salicylic acid |
| DES | desulfhydrase | OR | oxidordeuctase |
| DHA | docosahexaenoic acid | SCS | sulfocysteine synthase |
| DHAR | dehydroascorbate reductase | SnRK2.6 | snf1-related protein kinase 2.6 |
| EC | enzyme commission number | SiR | sulfite reductase |
