# Supplementary figures and images for: From odor to order: unveiling the crucial role of hydrogen sulfide in plant life
Source: Hortic Res. 2025 Oct 17;13(1):uhaf273. doi: 10.1093/hr/uhaf273 (PMC12881861; doi:10.1093/hr/uhaf273)

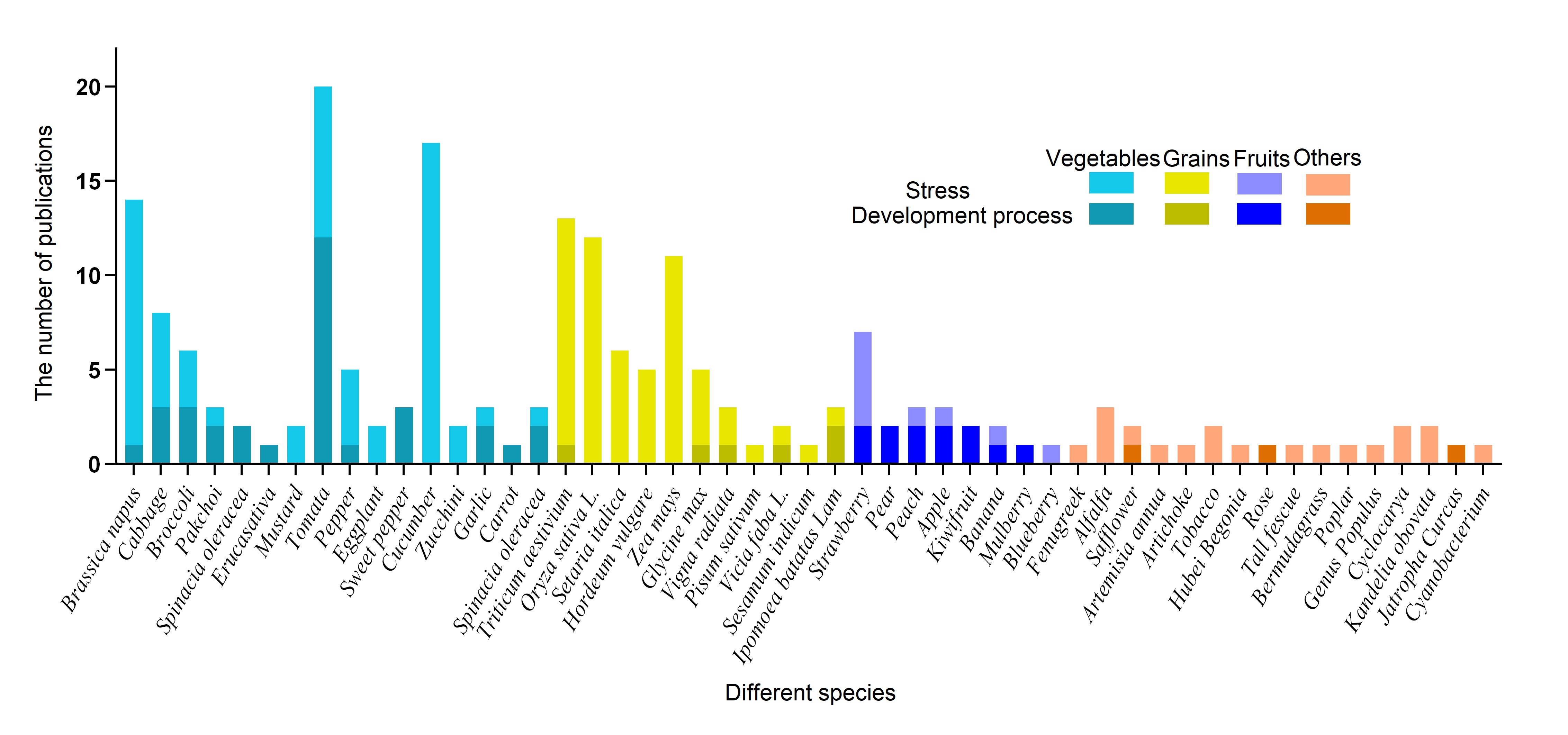

Supplement: Web_Material_uhaf273 [file web_material_uhaf273.zip › FIG S1.jpg]
